# Treating Postpartum Depression: What Do We Know about Brexanolone?

**DOI:** 10.3390/diseases9030052

**Published:** 2021-07-12

**Authors:** Muneeza Ali, Alifiya Aamir, Mufaddal Najmuddin Diwan, Hashir Ali Awan, Irfan Ullah, Muhammad Irfan, Domenico De Berardis

**Affiliations:** 1 Department of Internal Medicine, Dow Medical College, Karachi 74200, Pakistan; muneeza1998@gmail.com (M.A.); alifiya.aamir521@gmail.com (A.A.); mufdiwan@gmail.com (M.N.D.); hashiraliawan@gmail.com (H.A.A.); 2 Kabir Medical College, Gandhara University, Peshawar 25000, Pakistan; irfanullahecp2@gmail.com; 3 Department of Internal Medicine, Hayatabad Medical Complex, Peshawar 25000, Pakistan; irfanjamal2k18@gmail.com; 4 NHS, Department of Mental Health, Psychiatric Service for Diagnosis and Treatment, Hospital “G. Mazzini”, ASL 4, 64100 Teramo, Italy

**Keywords:** postpartum depression, brexanolone, pregnancy, safety, effectiveness

## Abstract

Postpartum depression (PPD) is defined as the onset of major depressive disorder in mothers, occurring during pregnancy or within 4 weeks post-delivery. With 7% of pregnancy-related death in the United States owing to mental health conditions, including PPD, and a global prevalence of 12%, PPD is a growing public health concern. In 2019, the Food and Drug Administration (FDA) approved brexanolone, an exogenous analog of allopregnanolone, as the first ever drug to be specifically indicated for treating patients with PPD. This approval was preceded by an open-label study and three randomized placebo-controlled trials, each assessing the safety, tolerability, and efficacy of brexanolone, using mean Hamilton Rating Scale for Depression (HAM-D) score reduction as the primary outcome. In each randomized controlled trial, the drug was administered as an intravenous infusion given over 60 h. Enrolled participants were followed up on days 7 and 30 to evaluate the sustained effect. A statistically significant reduction in mean HAM-D score compared to placebo was observed in all three studies, supporting brexanolone’s use in treating moderate-to-severe PPD. Therefore, this article attempts to briefly review the pharmacology of brexanolone, evaluate the latest available clinical data and outcomes concerning its use, reevaluate its position as a ‘breakthrough’ in managing PPD, and review the cost-related barriers to its worldwide standardized use.

## 1. Introduction

The Diagnostic and Statistical Manual (DSM-V) defines postpartum depression (PPD) as a recurrent or new onset of major depressive disorder (MDD) in mothers, with the episodes transpiring during pregnancy or within 4 weeks post-delivery [1]. However, in the realm of clinical practice and research, PPD is described as occurring from 4 weeks to 12 months following childbirth [2].

The question of what causes PPD has been countered by various etiological models. An important review by Yim et al. [3] divides them into biological, psychosocial, and integrative models. Therefore, PPD is considered as a biopsychosocial phenomenon [4].

Prevalence, symptoms, and impact of PPD are considered to be greatly influenced by a variety of psychosocial factors. Episodic and chronic stressors in a woman’s life have also been considered and extensively reviewed as independent and concurrent factors in the development of PPD [3]. A woman’s ability to understand and respond to stressful situations (called sense of coherence or SOC) has also been linked to developing PPD, with a high SOC acting as a protective factor [4]. The SOC, in turn, is also affected by various sociodemographic factors, such as work status, education, financial background, and marital status, depicting how they directly and indirectly affect a woman’s predisposition to PPD [4].

Notably, psychosocial factors play a major role in determining the prevalence of PPD in different parts of the world. Similarly, ethnocultural differences have also been reported to be responsible for varying prevalence and manifestation of other psychological disorders, such as post-traumatic stress disorder (PTSD), after birth [5]. Women from low- and middle-income countries are at a high risk of conditions pertaining to mental health and stressful life events due to predisposing factors, such as a history of abuse in childhood, intimate partner violence, and physical or emotional isolation. Additionally, prevailing socioeconomic conditions of the said regions are key elements in increasing risk of developing PPD due to fragile and dysfunctional healthcare systems; little to no awareness regarding the condition; and culture-exclusive factors, such as preference for a male firstborn. Consequently, an alarmingly high number of 1 in 5 women in such countries experience PPD [6].

Apart from psychosocial interplay, the biological pathophysiology of PPD is not completely ascertained. Several endocrine and genetic/epigenetic factors have been considered responsible for the appearance of symptoms. Most commonly, the endocrine model claiming rapid alterations in allopregnanolone concentration is widely believed to be the underlying etiology of PPD. A key metabolite of progesterone, allopregnanolone is a positive allosteric modulator of γ-aminobutyric acid (GABA) type A receptors. Its level in the maternal peripheral blood rises during the third trimester, followed by a rapid decline after childbirth. This decrease in levels of allopregnanolone in blood or cerebrospinal fluid (CSF) accounts for the increased risk of anxiety and depression [2,7].

Many comorbidities, such as gestational diabetes and preeclampsia, have been classified as predictors of PPD. In fact, Moreira et al. attempted to review state-of-the-art artificial intelligence (AI) and machine learning mechanisms to classify and predict the risk of psychological disorders in pregnant women based on their biomedical and sociodemographic characteristics [8]. It also highlighted emotion-aware computing that could enable the detection of behavioral changes, especially applicable to women with a high risk of developing PPD [8].

Genetic differences and epigenetic changes have also been noted as predictors of PPD severity and symptoms. Findings from various studies have been reviewed, illustrating the possible impact of heritable polymorphic differences in certain candidate genes, including those encoding for the COMT and MAO systems, estrogen receptor, oxytocin (and its receptor), and the glucocorticoid receptors, on occurrence and severity of PPD [3,9]. Multiple reviews also elucidate the potential importance of, and the increased susceptibility of women with PPD to, epigenetic changes [3], which further strengthens ‘the cross-talk between environment and genetics’ in the causation of PPD [9]. In 2018, Shorey et al. conducted a meta-analysis in which data from a multitude of studies representing all geographical regions were pooled, and it was observed that in a sample size of a total of 37,295 women, the global prevalence of PPD was 17%. Additionally, a statistically significant difference in prevalence between the regions was seen, with the Middle East having the highest prevalence of 26%, Europe having the lowest prevalence of 8%, and Asia having a prevalence of 16% [10]. Moreover, it is estimated that in the United States, the prevalence of women who experience PPD ranges from 8% to 20% [11]. The widely changing prevalence of PPD across the world, and even within certain regions, can potentially be attributed at least partially to genetic and epigenetic variations within and between populations.

In a 2018 report, it was reported that maternal mental health conditions, including PPD, lead to 7% of pregnancy-related deaths [12,13]. Furthermore, it is believed that approximately 40–80% of cases of PPD are moderate-to-severe in nature, warranting the need for focused medical intervention [14]. Women suffering from PPD may face wide-ranging negative implications, including suicidal ideations and death; unemployment; and infant morbidity associated with compromised mother–infant attachment, subsequently resulting in malnutrition during the first year of life. All the aforementioned factors amount to making PPD a growing public health concern [14].

According to the American College of Obstetricians and Gynecologists’ 2018 recommendations, the primary application of first-line treatment of PPD has relied on screening and pharmacologic intervention for the symptomatic management of depression and anxiety in addition to referral to mental healthcare providers [15,16]. The predominance of data in favor of selective serotonin reuptake inhibitors (SSRIs) and insufficient experimental data on adjunctive cognitive therapy or hormonal supplementation has often led to them being used as the default first-line drug therapy for PPD [14].

While the use of drug therapy is being evaluated as a mainstay in the management plan for PPD, it has been found that perinatal patients favor non-drug treatment [17]. A study conducted by Goodman et al., 2009, which included 509 pregnant women, concluded that 92% of the participants preferred individual psychotherapy and only 7% selected drugs as the first choice of treatment [18]. Similarly, a cross-sectional study was conducted on Israeli mothers by Simhi et al., 2019 [19], which compared preference to psychological treatment of mothers with and without PPD. This study concluded that mothers chose private mental health clinics and community centers as their preferred place of treatment, while the preferred mode of treatment was private meetings in an office with a professional. Moreover, the participants preferred personal psychotherapy intervention, mental health care professionals, and group interventions over interventions mediated by technology [19].

On 19 March 2019, however, the Food and Drug Administration (FDA) approved brexanolone, an aqueous formulation of allopregnanolone, as the first ever drug to be used specifically for the treatment of PPD [20]. The purpose of this article is to briefly review the pharmacology of brexanolone, evaluate the latest available clinical data and outcomes concerning its use, and review its position as a ‘break through’ in managing PPD worldwide.

## 2. Pharmacology

### 2.1. Mode of Action

Brexanolone, an exogenous analog of allopregnanolone and a neuroactive steroid, binds to five-unit transmembrane GABA type A receptors. Although the exact mechanism of action is still unknown, it is believed that, by binding to these receptors, the drug enhances the activity of GABA (inhibitory neurotransmitter). Subsequently, this results in reduced anxiety and depression-like symptoms. As a consequence of the inhibitory effects, the drug has side effects of sedation, manifesting as drowsiness and dizziness [12].

### 2.2. Dosage and Administration

Brexanolone is usually administered at inpatient facilities via a continuous intravenous infusion over a period of 60 h. The dosage per hour is gradually increased from 30 µg/kg/h to a maintenance dose of 90 µg/kg/h till 52 h, before being tapered off back to 30 µg/kg/h by 60 h. The specific dosage divided into hourly time periods is provided in Table 1 [21].

### 2.3. Pharmacokinetics

Brexanolone, having a half-life of nearly 9 h, has three major inactive metabolites with a total plasma clearance of 1 L/h/kg and equal amounts of excretion in the urine and feces. The drug undergoes considerable non-cytochrome P450 enzyme-mediated hepatic metabolism via keto reduction, glucuronidation, and sulfation, rendering the use of oral analog of allopregnanolone in clinical settings inefficacious and a resultant low oral bioavailability [22]. Furthermore, to date, no drug interaction has been reported apart from drug–drug interactions with CNS depressants and antidepressants, owing to the sedating adverse effects of brexanolone [12,23].

## 3. Review of Clinical Trials

As of now, a search of the literature for empirical evidence of brexanolone’s clinical assessment yielded three separate studies, consisting of a total of three randomized control trials (RCTs, Table 2) [14,21] and one proof-of-concept study [24].

### 3.1. Proof of Concept Study

The efficacy of brexanolone was assessed via a first-of-its-kind 35-day proof of concept study [24]. The study had a total of four partakers receiving a continuous 60 h infusion of brexanolone.

Those registered had a Hamilton Rating Scale for Depression (HAM-D) score of ≥20, had discontinued breastfeeding, experienced at least one MDD episode between the third trimester to 12 weeks after childbirth, and were admitted to a psychiatric unit after 2 and up to 20 weeks postpartum. Additionally, participants were allowed concomitant use of antidepressants if they had been in a stable condition for a minimum of two weeks prior to being enrolled into the study program.

The primary outcomes analyzed in the study were the safety and tolerability of the infusion, particularly considering any adverse effects during and after the administration of the drug. Moreover, baseline changes in HAM-D score were recorded to evaluate efficacy as a secondary outcome of the study. Other psychiatric outcomes were measured using the Generalized Anxiety Disorder 7-item scale (GAD-7) [25], the Patient Health Questionnaire (PHQ-9) [26], Clinical Global Impression-Improvement scale (CGI-I), and the Edinburgh Postnatal Depression Scale (EPDS) [27]. All four participants were monitored for safety and efficacy 14 h following the delivery of the last dose, and they were routinely followed up on days 11 and 35 after the study.

The study found that apart from sedation, no serious adverse effects were reported by the participants. Assessing the secondary outcome, from a baseline of 26.5 recorded in participants, the mean HAM-D score reduced to 4.8 at 12 h post-commencement and 1.8 at the end of infusion (60 h).

It is important to note that a substantial reduction in symptoms through the notable fall in HAM-D and EPDS scores was recorded. Despite the limitations of this study, such as having a small sample size, an open-label design, and an assessment time that was not sufficiently extensive, this was a landmark study carried out to preliminarily test the efficacy of brexanolone in treating PPD specifically, indicating that it was generally well tolerated. Kanes et al. therefore concluded that this study may potentially prove to be a significant milestone in identifying brexanolone as a more focused and targeted pharmacological treatment for PPD and pave the way for a greater understanding of its role [24,28].

### 3.2. Phase II Trial

Accordingly, in 2017, a double-blind, multicenter, parallel-group, randomized placebo-controlled trial was conducted by Kanes et al. [21]. A total of 21 women between the age of 18 and 45 years were enrolled into the study and were randomly divided into two groups: one to receive brexanolone, consisting of 10 participants, and the other one, acting as a control, to receive a placebo, consisting of 11 participants.

**Figure 1:**
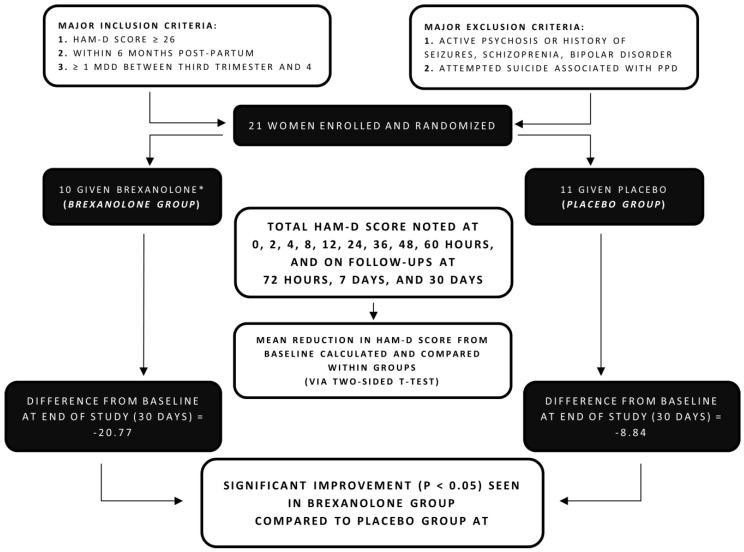
Flowchart summarizing methods and findings of randomized controlled trial by Kanes et al. 2017 [21].

Figure 1 attempts to briefly outline the method and findings of this trial and shows the major inclusion and exclusion criteria on which the patients were enrolled. Similar to the proof-of-concept study, participants were allowed concomitant use of antidepressants but only if they had been stable for a more extended period—a minimum of 30 days—prior to being enrolled into the study program.

The RCT lasted 30 days, during which a continuous infusion of brexanolone was given for 60 h (for dosage, refer to Table 1) initially, after which patients were assessed and followed up at 72 h post-commencement on infusion, then further on the 7th and 30th days. The salient features and findings investigated by this RCT are briefly provided below:

#### 3.2.1. HAM-D Scores:

HAM-D score was majorly used to evaluate primary outcome of an observed reduction in symptoms. On average, a reduction of 20.97 points from a baseline of the mean HAM-D score was reported in the brexanolone group compared to a reduction of 8.8 points in the control group at the end of infusion (60 h). Performing a two-sided t-test revealed that the brexanolone group saw significantly greater improvement than the placebo group at the end of the infusion and even further at follow-ups on the 7th and 30th days (Table 2).

#### 3.2.2. Remission, Response, and Other Parameters

On the other hand, the secondary outcomes were to ascertain how many participants achieved ‘remission’ (a drop in HAM-D score to 7 or below), how many participants achieved ‘response’ (a drop in HAM-D score to ≥50% of baseline), the Montgomery–Asberg Rating Scale (MADRS) total score [29], major depression, and changes in CGI-I score.

The study reported that 70% of the brexanolone recipients achieved ‘remission’, which was significantly greater than the placebo group. In addition, 70% of the brexanolone recipients also depicted ‘response’, as their HAM-D scores were half (or lower) than their baseline scores. Along with the HAM-D scores, a significant improvement in symptoms was also observed using the MARDS and CGI-I response.

#### 3.2.3. Safety, Sedation, and Adverse Effects:

In order to monitor the safety and tolerability of brexanolone, vitals and echocardiogram (ECG) changes from baseline were monitored and any occurrence of adverse effects was recorded. In total, 40% of the patients in the brexanolone group reported an occurrence of adverse events, on the contrary, a much higher number (72.7%) of adverse events was recorded in the placebo group.

Furthermore, developing suicidal ideation assessment was carried out with the Columbia-Suicide Severity Rating Scale [30], and reports of sedation were evaluated with the Stanford Sleepiness Scale [31]. Improvements were noted in suicide severity, and a further worsening of suicide ideation was not recorded in either group. The mean Stanford Sleepiness Scale scores were alike (2.7 in the brexanolone group compared to 2.6 in the placebo group).

This study provided the first placebo-controlled clinical trial to support the use of allopregnanolone analog in the treatment of PPD; however, this trial was limited by a small sample size, a strict severe PPD definition of HAM-D ≥ 26, possible respondent fatigue in HAM-D assessment, and the short follow-up period (30 days) [12,21,28]. In contrast to the studies assessing first-line drug treatment SSRIs and other widely used antidepressant drugs, such as serotonin-noradrenaline reuptake inhibitors (SNRIs) and tricyclic antidepressants, the aforementioned trial had a sizable effect size of 1.2 [32,33].

### 3.3. Phase III Trial

Meltzer-Brody et al. subsequently conducted two double-blind, randomized, placebo-controlled, phase III trials to further assess the safety and efficacy of brexanolone [14]. Both trials enrolled 246 women between the age of 18 and 45 (of which 138 were randomly assigned to study 1 and 108 to study 2). Of the participants assigned to each study, 16 of them from study 1 and 4 from study 2 were not given the infusion due to various reasons and hence were not analyzed [14].

**Figure 2:**
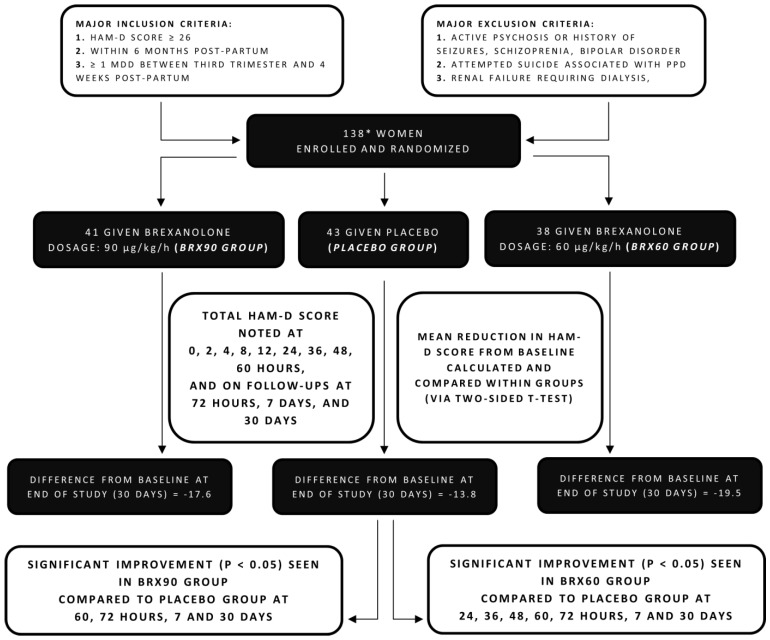
Flowchart summarizing methods and findings of randomized controlled trial (study 1) by Meltzer-Brody et al. 2018. * 138 participants were enrolled, of which 16 were not included in analysis for various reasons [14].

For study 1, patients were randomly divided to either receive brexanolone injection at 90 μg/kg/h (group referred to as BRX90), brexanolone injection at 60 μg/kg/h (group referred to as BRX60), or a placebo infusion. In this study, the inclusion criteria required the participants to have a baseline HAM-D score of 26 or more. Figure 2 summarizes the notable features and findings of this study.

**Figure 3:**
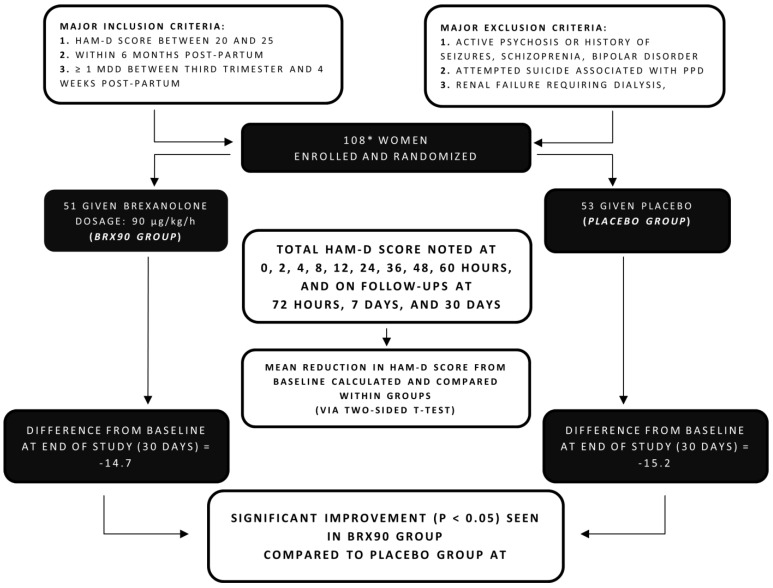
Flowchart summarizing methods and findings of randomized controlled trial (study 2) by Meltzer-Brody et al. 2018. * 108 participants were enrolled, of which 4 were not included in analysis for various reasons [14].

For study 2, patients were also randomly divided to receive either brexanolone injection at 90 μg/kg/h (group referred to as BRX90) or a placebo infusion. However, unlike the first study, study 2 specified the eligibility criteria of participants as having a baseline HAM-D score of 20–25. The important methods and primary findings are given in Figure 3.

Both studies, similar to Kanes et al.’s RCT [21], continued for 30 days with a continuous 60 h infusion of brexanolone or a placebo administered to participants, with monitoring at specific intervals till hour 72 (post-infusion) and follow-ups on days 7 and 30. The notable outcomes are specified below:

#### 3.3.1. HAM-D Scores:

The primary outcome measured in both the studies was the least squares (LS) change from baseline in mean total HAM-D score (particularly at 60 h post-commencement).

In study 1, LS mean reduction in HAM-D score at the end of the 60 h infusion was 19.5 in the BRX60 group and 17.7 in the BRX90 group, which were both significantly greater than the placebo group, recording more improvement in brexanolone-receiving groups. Continuing this trend, a significantly higher reduction in HAM-D total scores from baseline at day 30 was observed in the BRX60 and BRX90 groups in contrast to the placebo group (Table 2).

In study 2, the LS mean reduction in HAM-D score was obtained for the BRX90 group and compared with the group receiving placebo. At 60 h post-commencement, the BRX90 group had an LS mean reduction of 14.6, which was significantly more than the placebo group. Notably, contrary to study 1, no significant reduction in HAM-D was noted in study 2 partakers on day 30 when compared to the placebo (Table 2).

#### 3.3.2. Remission, Response, and Other Parameters

In comparison to the placebo groups, a greater fraction of patients receiving BRX60 and BRX90 reported remission (defined by Kanes et al. [21] as mentioned above) of statistical significance in both the studies. At the end of the 60 h infusion period, 51% of participants in the BRX60 group of study 1 and 61% of participants in the BRX90 group of study 2 reached remission. The study reports that the ratio of participants achieving response was similar for all groups receiving brexanolone across both studies with a significant difference from placebo groups at various timepoints throughout the 30-day period.

CGI-1 response and a change in total score from the baseline of MADRS, EPDS, PHQ-9, and GAD-7 were also evaluated in the two studies for all groups. The results obtained via analysis of the HAM-D scores were further emphasized by a significantly higher ratio of brexanolone-receiving groups achieving a CGI-I response compared to placebo groups.

#### 3.3.3. Safety, Sedation, and Adverse Effects

Safety and tolerability were assessed by monitoring vitals and ECG, recording the occurrence and frequency of any adverse events, and the Columbia Suicide Severity Rating Scale scores, utilized to determine any suicidal ideation and risk.

The drug was generally well tolerated by the participants with headache being the most common adverse effect, with its prevalence ranging from 15% to 18% of the participants in the brexanolone-receiving groups in both studies. A greater number of brexanolone receivers reported episodes of dizziness and somnolence paralleled to the placebo group. In study 1, 18% of the patients receiving BRX60, 5% receiving BRX90, and 7% receiving placebo reported somnolence. In study 2, 8% of the participants in the BRX90 group reported somnolence, which was double that of the participants in the placebo group (4%). Other noted adverse effects were dry mouth, fatigue, nausea, and infusion site pain.

With the limitation of this study representing a patient population with severe and moderate PPD, the notable exclusion of women with mild PPD thus requires the need for more empirical data in order for outcomes to support generalized brexanolone use for a wider population [14,28].

## 4. SSRIs and Brexanolone

Typically, moderate-to-severe PPD is managed using selective serotonin reuptake inhibitors (SSRIs). A total of four open-label [34,35,36,37] and eight RCTs [38,39,40,41,42,43,44,45] have evaluated SSRIs with assessment indicating mixed results in terms of efficacy and tolerability in using them as antidepressants to treat PPD. Furthermore, a Cochrane review on three studies comparing SSRIs with placebos for PPD was conducted by Molyneaux et al. [46], which reported that patients did exhibit response and remission to the treatment [47].

In 2019, Cooper et al. conducted a meta-analysis to compare the efficacy of brexanolone infusion with SSRIs for treating PPD. Due to the lack of RCTs comparing both drug therapies, an indirect treatment comparison (ITC) [48] approach was adopted. Using the data from available studies, the HAM-D score was selected, as it is regarded as the ‘gold standard’ for measuring outcomes relating to depression. Since EPDS is regularly used to screen for PPD in clinical practice, it was also chosen as an outcome.

Randomized and controlled studies with at least one pharmacological arm and outcome in the form of two parameters, HAM-D and/or EPDS, were selected for this comparison. Matching-adjusted indirect comparison (MAIC) results indicated greater effectiveness of BRX90 compared to SSRIs. Furthermore, using the MAIC-adjusted Bucher ITC and standard network meta-analysis (NMA), it was deduced that not only was brexanolone’s efficacy rapid, but it also had sustained efficacy compared to the other group.

The authors of this study, however, did point out the lack of evidence in determining the impact of the variable severity of depression of the study participants on the ITC results. Additionally, the placebo groups to which brexanolone was matched/adjusted was ‘subjective’; therefore, a difference may lead to a change in results [49].

## 5. Conclusions

Brexanolone is being hailed as a ‘breakthrough’ medication for the treatment of PPD [50]. As highlighted in this review, the positive outcomes with regard to the clinical use of the drug obtained from the three RCTs gave extensive evidence in favor of the safety, tolerability, and efficacy of brexanolone. Consequently, it prompted the FDA to give brexanolone a ‘priority review’ and ‘breakthrough therapy’ classification, which ultimately led to its approval [20].

Nevertheless, the drug has its shortcomings. The continuous infusion, the need for an inpatient facility, requiring continuous pulse oximetry monitoring, and side effects such as sedation leading to the discontinuation of this treatment all add to the challenges of brexanolone becoming a real-world practical treatment for PPD [51]. Further adding to the barriers is the total cost of USD 34,000 (USD 7450 per vial and about 4.58 vials on average), excluding the inpatient facility cost [50,51]. This makes its use as the treatment of choice cost 36 times more in contrast to mainstream therapy. In addition, there is still a lack of data exploring the long-term efficacy of this drug, which, if inefficacious beyond the 30-day sustained affect (analyzed in all three RCTs), may incur greater costs and make it considerably more difficult to administer [52]. All the aforementioned elements would contribute to causing a greater burden for patients and facilities, especially in low- and middle-income countries, when opting for brexanolone as the choice of pharmacological therapy. However, despite these drawbacks, the rapid onset of this drug along with its sustained efficacy, especially in comparison to standard antidepressant SSRIs, offers some hope.

There is a pressing need to collect further evidence to safely utilize brexanolone in a wider patient population [50]. While some authors believe brexanolone will not unfavorably affect infants being breastfed by mothers undergoing treatment [22], the scarcity of clinical data supporting this claim along with all three RCTs excluding women who were breastfeeding prompts to expand our clinical trials. In addition, the RCTs included had a similar cohort with a small sample size, and the patient population did not include women experiencing mild PPD [7]. Importantly, to date, no clinical trial directly comparing the efficacy of brexanolone with other antidepressants has been published. Furthermore, the long-term safety and risk of developing worsening adverse effects over time after drug consumption have also not been conclusively evaluated [52].

This article is limited by a small amount of empirical data available to review and a lack of direct comparison with other drug-based regimens and non-drug therapies. As PPD is largely a biopsychosocial phenomenon, drug regimens such as brexanolone can be accompanied with cognitive and psychological therapy, therefore creating a more holistic treatment pattern addressing biomedical and psychosocial aspects of the condition. Notably, the lack of specific tools to assess PPD and the use of generic scoring methods may also limit our understanding of PPD, the efficacy of brexanolone in its treatment, and, in turn, the findings of this review. While the use of emotion-aware computing may act to fill this gap [8] and help us subjectively recognize and document PPD, we suggest focus be placed on developing and validating questionnaires focused solely on PPD and associated symptoms.

With oral formulation also being evaluated (SAGE-217 and ganaxolone) [47], further studies on the drug exploring these factors may potentially cause a drastic shift in brexanolone’s place in psychiatry, proving to be extremely beneficial in improving the quality of life of millions of women across the globe.

## Figures and Tables

**Table 1:** Dosage of Brexanolone.

Time Frame	Dosage (In µg/kg/h)
0 to 4	30
4 to 24	60
24 to 52	90
52 to 56	60
56 to 60	30

Table 1: Dosage of brexanolone during the 60-h infusion as described by Kanes et al. [21].

**Table 2:** Significant results from the three available RCTs.

Study/Trial	Brexanolone Dosage (μg/kg/h)	Number of Participants (n)	HAM-D LS Mean Change from Baseline at 60 h (*p*-Value *)	HAM-D LS Mean Change from Baseline at 72 h (*p*-Value *)	HAM-D LS Mean Change from Baseline at 7 d (*p*-Value *)	HAM-D LS Mean Change from Baseline at 30 d (*p*-Value *)
Kanes et al. 2017 [21]	(Table 1)	10	−21.0 (0·0075)	−21.0 (0.0078)	−21.0 (0.0038)	−20.8 (0.0095)
Meltzer-Brody et al. 2018—study 1 [14]	60 (BRX60)	38	−19.5 (0.0013)	−19.7 (0.0046)	−17.4 (0.0288)	−19.5 (0.0044)
	90 (BRX90)	41	−17.7 (0.0252)	−17.2 (0.1389)	−14.9 (0.3799)	−17.6 (0.0481)
Meltzer-Brody et al. 2018—study 2 [14]	90 (BRX90)	51	−14.6 (0.0160)	−15.3 (0.0022)	−14.0 (0.0255)	−14.7 (0.6710)

Table 2: Brief summary of 3 RCTs conducted to evaluate brexanolone efficacy with Hamilton Rating Scale for Depression (HAM-D) least square (LS) mean change from baseline at four timepoints (60 h, 72 h, 7 days, 30 days) post-commencement. * *p*-values obtained via two-sided *t*-test and compared to placebo group(s) for each study and timepoint. *p* > 0.05 is considered significant.

## Data Availability

Not applicable.
